# Different Types of Vasculitis Complicated by Heparin-Induced Thrombocytopenia—Analysis of Four Cases and Literature Review

**DOI:** 10.3390/jcm12196176

**Published:** 2023-09-24

**Authors:** Adam Rytel, Mateusz Nowak, Monika Kukawska-Rytel, Katarzyna Morawiec, Stanisław Niemczyk

**Affiliations:** Department of Internal Diseases, Nephrology and Dialysis, Military Institute of Medicine—National Research Institute, 04-141 Warsaw, Polandkasia.morawiec123@gmail.com (K.M.);

**Keywords:** HIT, vasculitis, ANCA vasculitis, anti-GBM disease

## Abstract

Vasculitis and HIT have different etiologies, although both involve autoimmune mechanisms. Treatment of vasculitis often requires the use of an anticoagulant such as heparin, which can lead to the development of HIT and subsequent life-threatening complications. The analysis covered patients hospitalized in the Department of Internal Medicine, Nephrology and Dialysis in the period from September 2020 to March 2023. After analyzing the data, we selected four patients in whom vasculitis treatment was complicated by HIT. These included two patients with ANCA vasculitis and two patients with anti-GBM disease. We also described similar cases reported in the literature.

## 1. Introduction

Heparin-induced thrombocytopenia (HIT) is a life-threatening immunologic adverse effect of exposure to heparin. It is caused by antibodies that recognize complexes of platelet factor 4 and heparin (anti-PF4/H Abs). The main clinical manifestation of HIT is thrombosis, although thrombocytopenia is the predominant abnormality in laboratory tests [1]. There are two types of HIT, of which type I is a non-autoimmune reaction in the first two days after heparin administration and is not associated with an increased risk of thrombosis and is considered clinically insignificant. In contrast, type II, associated with severe thrombocytopenia and thrombosis, is considered clinically significant. HIT occurs in 0.5% to 1% of patients exposed to unfractionated heparin for more than 4 days (usually occurs 5–14 days after exposure) [2].

According to the 2012 Revised International Chapel Hill Consensus Conference, vasculitis is the inflammation of blood vessel walls. It is a heterogeneous group of pathologies that can be divided according to the size of the affected vessel [3]. In our nephrology department, we mainly treat small-vessel vasculitis which can be divided into two groups defined by the presence or absence of immunoglobulin complex deposition in the vessel wall. The group without deposition of immune complexes is usually associated with anti-neutrophil cytoplasmic antibodies (ANCA), while the groups associated with deposition of immune complexes include anti-glomerular basement membrane disease (anti-GBM) disease, cryoglobulinemic vasculitis, IgA vasculitis (IgAN) and hypocomplementemic urticarial vasculitis [4]. Since vasculitis is a rare disease, there is little data on individual complications during treatment. We decided to analyze the data available in the electronic database on patients hospitalized in our department whose treatment was complicated by HIT. Patients treated for vasculitis were over-represented in this group compared to other patients. The association between HIT and certain autoimmune diseases (for example, systemic lupus erythematosus, rheumatoid arthritis) has been reported in many papers [5,6]. However, there is a lack of similar studies covering vasculitis, which is also a group of autoimmune-related diseases.

The aim of this study was to analyze the clinical presentation and laboratory findings in a group of patients diagnosed with vasculitis who developed HIT during hospitalization and also to discuss similar cases reported in the literature.

## 2. Materials and Methods

The analysis covered patients hospitalized in the Department of Internal Medicine, Nephrology and Dialysis of the Military Institute of Medicine—National Research Institute in the period from September 2020 to March 2023. The source of the data was the electronic database of our hospital. Our goal was to search for patients with HIT diagnosed during hospitalization. In this group, we selected cases in which HIT was diagnosed during treatment for small-vessel vasculitis. In all patients, HIT was confirmed by the presence of anti-PF4/H Abs.

The literature review was performed with the use of the MEDLINE database/archive via the PubMed and Google Scholar databases in order to identify documented cases of small-vessel vasculitis complicated by heparin-induced thrombocytopenia. The search algorithm was conducted using the following terms in order to locate all relevant records: (vasculitis or small-vessel vasculitis or ANCA vasculitis or anti-GBM disease or AAV or IgA vasculitis or cryoglobulinemic vasculitis) and (heparin-induced thrombocytopenia or HIT). We excluded case reports in which HIT was not confirmed by anti-PF4/H Abs testing.

## 3. Results

**Figure 1:**
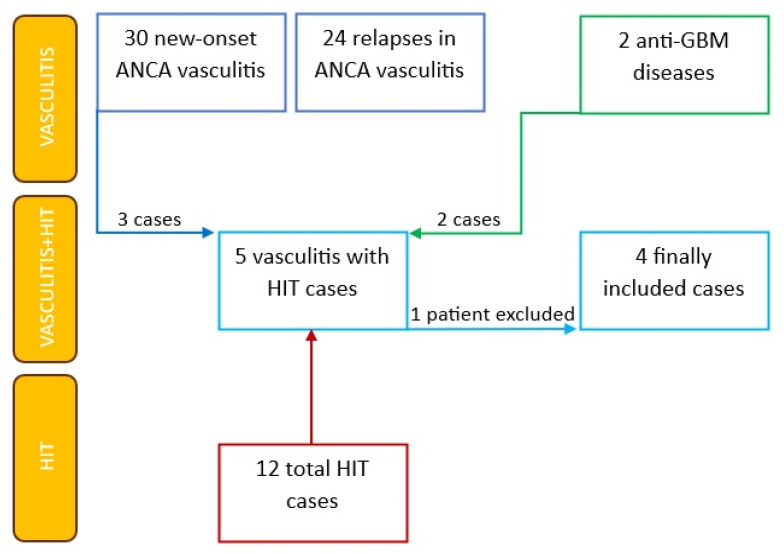
All cases of vasculitis and HIT during the selected period in our department and the final selected cases.

A total of 12 cases of HIT were diagnosed in our department during the given time period. In the same time frame, the department hospitalized 54 patients with ANCA vasculitis who had been started on treatment or had treatment re-initiated due to relapse. Vasculitis was newly diagnosed in 30 patients (56%), while 24 patients (46%) were diagnosed with relapse. In the analyzed period, 2 patients were diagnosed with anti-GBM disease, while 5 out of 12 patients (42%) diagnosed with HIT were hospitalized for vasculitis. Approximately 2000 patients were hospitalized in the department during the period stated, of whom only 7 were diagnosed with HIT (Figure 1).

Of the five above-mentioned patients, we excluded one ANCA vasculitis male patient diagnosed with HIT (he was transferred from another center where HIT was suspected and heparin had been discontinued earlier). Ultimately, all criteria were met in the analyzed group by two women and two men, with a mean age of 60 years (ranging from 39 to 74 years). Two patients were diagnosed with vasculitis with pANCA antibodies. Only two patients hospitalized in the indicated period were diagnosed with anti-GBM disease, both were diagnosed with HIT in the course of treatment.

**Figure 2:**
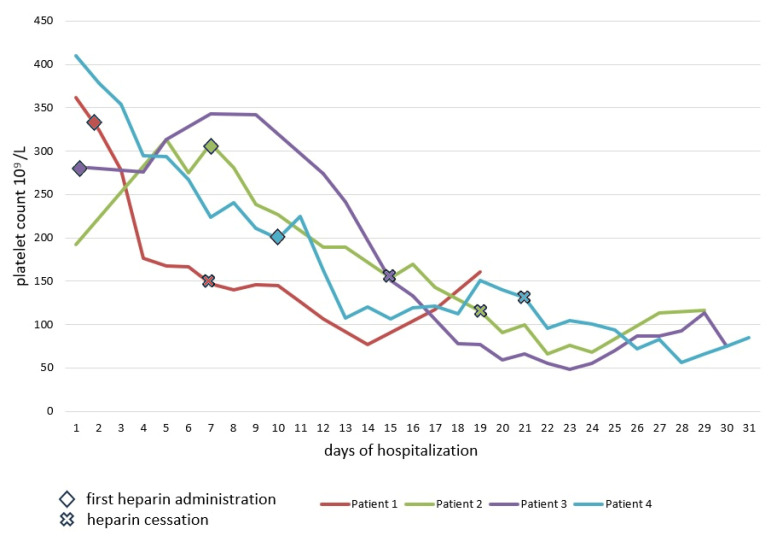
Decrease in platelet count in patients with HIT.

Although unfractionated heparin (UFH) is used much less frequently than low-molecular-weight heparin (LMWH) in everyday practice, it was responsible for the development of three out of four HIT cases. All cases were related to the administration of UFH during therapeutic plasma exchange (TPE) procedures. LMWH, which was responsible for the development of HIT in one patient, was administered as a prophylaxis of venous thromboembolism. The average time to 50% PLT reduction was 11 days (Figure 2).

Below we present four described clinical cases.

### 3.1. Case 1

A 71-year-old female patient with type 2 diabetes was initially admitted to another hospital due to increased nitrogen retention parameters, elevated inflammatory markers and signs of acute kidney injury (creatinine 1.9 mg/dL, normal in previous tests). The patient reported coughing and hemoptysis for several days before arriving at the hospital. Chest computed tomography (CT) showed diffuse ground-glass opacities and bilateral consolidative opacities. In the autoimmune panel that was performed, pANCA antibodies were positive. The tests performed did not indicate the presence of malignancy. Before starting treatment, infection was excluded. Taking into account the clinical picture and the tests performed, a diagnosis of microscopic polyangiitis was made. Intravenous steroid therapy (methylprednisolone pulses) was started. Due to the symptoms of infection, antibiotic therapy was administered in the days following the initiation of treatment, and a blood transfusion was given due to anemia. Respiratory failure developed during hospitalization; therefore, passive oxygen therapy was initiated. The patient was transferred to our department for further treatment. The initiated treatment was continued and the first infusion of cyclophosphamide was administered. A total of three TPE procedures were performed; hemoptysis stopped after the first TPE treatment. On the sixth day after the first heparin administration during TPE procedure, a 50% decrease in platelet count was observed. Due to suspected HIT, fraxiparine was used for the last TPE procedure. Anti-PF4/H Abs were present in the serum and HIT was diagnosed. The patient was discharged home after significant clinical improvement. A further treatment regimen inducing remission of the disease was established. On a follow-up chest CT scan, a gradual withdrawal of opacities was described.

### 3.2. Case 2

A 58-year-old male patient with a history of ulcerative colitis and bronchiectasis was admitted to our department due to a significant increase in renal parameters (creatinine 6.96 mg/dL, urea 141.1 mg/dL (normal range 15–43 mg/dL)) and normocytic anemia (hemoglobin 10.1 g/dL (12–16 g/dL for male patients)). Moreover, the patient reported nocturia, darkening of the urine for approximately 3 months, pain in the left lumbar region radiating to the front of the abdomen, persisting for approximately 2–3 weeks, weakness and worsened exercise tolerance. Due to the pain, he used non-steroidal anti-inflammatory drugs. In the general urine test, proteinuria and dysmorphic erythrocytes were observed. Daily proteinuria was 2.6 g. The anti-GBM antibodies titer was found to be positive and ANCA antibodies were negative. A chest CT showed ground-glass opacities and bronchiectasis. A diagnostic kidney biopsy was performed with a non-diagnostic result. Given the typical clinical picture and the anti-GBM antibodies present, we diagnosed anti-GBM disease and started treatment immediately. Immunosuppressive treatment with methylprednisolone pulses and oral prednisone was started. A total of 11 TPE procedures were performed until anti-GBM titers were undetectable, initially with the use of heparin. Control laboratory tests showed a gradual decrease in the number of platelets. Due to the suspicion of type 2 HIT, the anticoagulant treatment used during the procedures was changed—fondaparinux was added to the therapy and anti-PF4/H Abs were tested with a positive result. In the following days, the patient was included in the cyclophosphamide treatment program and another hospitalization was planned for a second pulse. Despite the administered treatment, the parameters of renal function remained increased.

### 3.3. Case 3

A 39-year-old male patient, not treated for chronic diseases, with a history of alcohol abuse, nicotinism and occupational exposure to silica dust, was transferred to our department from another center. The patient was admitted to the first hospital for treatment of acute kidney injury with features of nephritic syndrome and changes in the lungs. Renal biopsy showed glomerulonephritis with the presence of crescentic glomerulonephritis. Chest HRCT showed interstitial inflammatory changes covering approximately 50% of the lung tissue. The presence of positive pANCA antibodies was determined. The presence of the SARS-CoV-2 virus was found in the nasopharyngeal swab. Broad-spectrum antibiotic therapy was initiated. The patient was transferred to our department in order to continue the treatment of vasculitis. Increased values of creatinine and urea, hematuria with proteinuria, and anemia persisted during examinations. Treatment with steroids and intravenous pulses of cyclophosphamide was started. Conservative treatment of acute kidney injury was continued, but no improvement was achieved. It was decided to implement renal replacement therapy with hemodialysis using heparin anticoagulation. In the control morphology, due to persistent anemia and thrombocytopenia, HIT was suspected; therefore, anti-PF4/H Abs were marked, with a positive result. The heparin was replaced with fondaparinux, and the follow-up examinations showed an increase in the level of platelets. Despite the implemented treatment, the kidney function did not improve; therefore, the patient was included in the long-term hemodialysis program.

### 3.4. Case 4

A 74-year-old female patient treated for hypertension, type 2 diabetes, anemia and gout was urgently admitted to the department due to acute kidney injury and respiratory failure. On the day of admission, verbal contact with the patient was severely impeded, the interview with the family showed that the patient had been feverish for a week, urinated limited amounts of urine and vomited. In laboratory tests, significantly elevated kidney function parameters were observed: creatinine 7.2 mg/dL and urea 193 mg/dL, as well as high inflammatory parameters, acidosis, hyperkalemia, hyperphosphatemia and hypocalcemia. A chest X-ray showed signs of chronic pulmonary congestion. Empirical antibiotic therapy was started, which was then converted due to persistently high inflammatory markers, impaired consciousness and anuria. The patient developed gastrointestinal bleeding; therefore, a hemostatic clip was placed in the duodenum and parenteral nutrition was introduced. As a result of a failed attempt at conservative treatment of acute renal failure, the patient required hemodialysis during hospitalization. The presence of pANCA and anti-GBM antibodies was observed in the tested autoimmune panel. Then, the therapy was extended with pulses of methylprednisolone followed by oral prednisone and TPE procedures with the use of unfractionated heparin were initiated. After the implementation of treatment, progressive thrombocytopenia raised the suspicion of HIT, which was confirmed by a positive result for the presence of anti-PF4/H Abs. Heparin was changed to fondaparinux with good effect. During the hospital stay, the patient required a total of 12 TPE procedures until anti-GBM antibodies were not observed in laboratory tests. The first pulse of cyclophosphamide was administered, and another administration was scheduled two weeks later. The respiratory stable patient was discharged home, but due to the lack of improvement in kidney function during the hospital stay, a long-term hemodialysis catheter was inserted and the patient’s care was taken over by the local dialysis center. 

We present the above cases in a table together with the cases described in the literature (Table 1).

## 4. Discussion

Vasculitis and HIT have different etiologies, although both involve autoimmune mechanisms. These two distinct conditions can coexist in a patient, and their simultaneous occurrence can lead to complex clinical scenarios. HIT is associated with the presence of antibodies that recognize complexes of platelet factor 4 and heparin [18]. Development of anti-neutrophil cytoplasmic antibodies in ANCA vasculitis is associated with the loss of immunological T-cell and B-cell tolerance to one of two neutrophil proteins: leukocyte proteinase 3 or myeloperoxidase [19]. Anti-GBM disease is caused by the development of pathogenic antibodies targeting autoantigen expressed in the basement membranes [20]. The pathogenesis of both vasculitis and HIT involves dysfunction of the humoral system and the production of pathological autoantigens, but no data are available on the link between these diseases. There are studies that indicate that HIT is more common in people with certain autoimmune diseases, but they do not include its association with vasculitis [6,21]. 

Thrombosis is the most common and dangerous complication of HIT, it occurs in 20–64% of diagnosed patients and it is fatal in 6–26% of cases [22]. HIT is associated with the presence of antibodies against complexes formed from heparin and platelet factor 4, which have the ability to activate platelets, vascular endothelial cells and monocytes. This leads to a hypercoagulable state that causes arterial and venous thrombosis. Despite differences in pathogenesis, vasculitis can also lead to thrombosis. In vasculitis, there is an increased likelihood of thrombosis both in the acute phase of the disease and in remission. The cause is complex and not fully understood, but endothelial injury appears to play a major role [23]. Both vasculitis and HIT are associated not only with an increased risk of thrombosis, but also with an increased risk of bleeding. In a study focusing on bleeding in patients with HIT, clinically significant bleeding was described in 41% of patients [24]. Bleeding into the alveoli is a potentially life-threatening manifestation of vasculitis, but bleeding involving other systems has also been reported of as the first sign of the disease [25]. Co-occurring symptoms in both diseases can lead to wrong therapeutic decisions and delayed diagnosis, as well as intensification of symptoms, which can result in a worsening of the patient’s prognosis. Delayed diagnosis is associated with a worsened prognosis [26].

The group we described consisted of four patients in whom heparin administration during treatment was complicated by the occurrence of HIT, confirmed by an enzyme-linked immunosorbent assay (ELISA) test. In all of them, it was the first episode of the disease, and no case occurred during remission treatment. Among the patients we described, three of four developed HIT after the administration of unfractionated heparin during the plasmapheresis procedure. It has been shown that the use of unfractionated heparin increases the risk of HIT in certain groups of patients. In patients undergoing surgery, the use of UFH is by far a more common cause of HIT than in LMWH (risk 2.6% vs. 0.2%) [27], while, in other clinical circumstances, the situation is not clear [28,29]. During the TPE procedure, anti-PF4/H Abs are removed. Although TPE is not routinely used in the treatment of HIT, it can be used, among others, in cases of severe symptoms or in the need for urgent surgery [30]. In two of the patients we described, HIT occurred during a cycle of TPE treatments (in one patient, HIT was diagnosed after the cycle ended), yet we observed a decrease in platelet count by more than half on days 10 and 11 from a positive anti-PF4/H Abs immunoassay. This confirms the results of a study that suggested that anti-PF4/H Abs are detected in serum despite TPE treatments [31]. However, no thrombosis or bleeding that could be associated with the development of HIT was observed among the described patients. Two patients experienced alveolar hemorrhage; however, that had its onset prior to heparin exposure and was caused by underlying disease activity. The above-mentioned patients did not develop symptoms of HIT. The main way to avoid symptoms is to quickly link the developing thrombocytopenia to the development of HIT and change the anticoagulant [32]. Fraxiparine was used as a substitute for heparin, which is related to the lack of availability of other substitutes (i.e., argatroban, danaparoid) [33]. Larger, multicenter studies are needed to determine whether heparin use during the treatment of vasculitis is associated with an increased incidence of HIT. The potential pathogenesis of the increased occurrence of HIT in the autoimmune disease population also needs to be investigated. 

### 4.1. Literature Review

The amount of data in the literature on the co-existence of these two diseases is limited; after searching online databases, we found a total of 12 cases of vasculitis and HIT co-occurrence. Table 1 shows all cases described in the literature, including those described in this article. A total of six cases associated with ANCA vasculitis can be found (three cases were associated with antibodies against PR3 and three cases were associated with antibodies against MPO). The mean age of the patients was 74 years, with a slight male predominance (4/6). All were treated with corticosteroids, and most also received cyclophosphamide (4/6). All were on renal replacement therapy, one patient was on continuous venovenous hemofiltration; the remaining five patients were on intermittent hemodialysis. Six patients with anti-GBM disease were reported, with a significant male predominance (5/6) and significantly lower mean age (58 years) than in the ANCA antibody group. All patients with anti-GBM disease received glucocorticosteroids, were on therapeutic plasma exchange and intermittent hemodialysis, and most were treated with cyclophosphamide (4/6). In both groups, the time to a 50% decrease in platelet count after heparin exposure was 10 days.

We excluded from the analysis cases that were not confirmed by the anti-PF4/H Abs test. Among patients in whom the type of heparin used was specified, the overwhelming majority were patients treated with UFH (seven out of nine cases). Including our cases, 10 out of 13 cases reported in the literature were related to UFH use. This corresponds with studies that show a higher risk of HIT after the use of UFH compared to LMWH. Due to the rarity of vasculitis, there is a lack of research on the association between heparin and the occurrence of HIT in this condition. The group of patients with anti-GBM disease was significantly younger—58 years vs. 74 years. All of the patients required renal replacement therapy and all also used glucocorticosteroids. The time to a decrease in platelet count of approximately 50% in both groups was 10 days. 

### 4.2. Study Limitations

Our study has several potential limitations. The group analyzed in the department was small, with only 56 patients treated for vasculitis. This limits the ability to collect more data on such a rare complication as vasculitis. Another limitation is the retrospective nature of the study, which carries a selection bias. This work also does not include an analysis of HIT cases in patients without vasculitis.

## 5. Conclusions

The heterogeneity of symptoms and the lack of access to all diagnostic methods for both vasculitis and HIT can lead to prolonged hospitalization. It is important to keep in mind the possibility of co-occurrence of both conditions, since patients with new-onset vasculitis are often exposed early to heparin, either as thromboprophylaxis or during dialysis. Multicenter studies are needed to further investigate the topic we are discussing.

## Figures and Tables

**Table 1:** Overview of articles reporting coexisting cases of ANCA vasculitis/anti-GBM disease and HIT, including cases from this article.

Author and Year	Sex	Age	Vasculitis Type	Heparin Type	Days to 50% Drop in Platelet Count after First Exposure to Heparin	Treatment
**ANCA vasculitis**						
Roe et al., 1998 [7]	M	65	PR3	UFH	9 days	GCs, CP, HD
Balasubramanian et al., 2005 [8]	M	52	PR3	unspecified heparin	-	GCs, CP, CVVH, TPE, ECMO
Kaneda et al., 2009 [9]	F	91	MPO	UFH	13 days	GCs, HD
Thong et al., 2013 [10]	M	71	PR3	UFH and dalteparin	7 days	GCs, CP, HD
Nonaka et al., 2019 [11]	F	87	MPO	UFH	8 days	GCs, HD
Furuto et al., 2021 [12]	M	76	MPO	UFH	14 days	GCs, CP, HD
Case 1 *	F	71	MPO	UFH	6 days	GCs, CP, TPE
Case 3 *	M	39	MPO	LMWH	14 days	GCs, CP, HD
**anti-GBM vasculitis**						
Thong et al., 2013 [10]	M	80	anti-GBM	UFH and dalteparin	8 days	GCs, CP, TPE, HD
Mandai et al., 2011 [13]	M	40	anti-GBM + MPO	UFH	5 days	GCs, CP, TPE, HD
Miki et al., 2012 [14]	M	69	anti-GBM	unspecified heparin	15 days	GCs, TPE, HD
Micarelli et al., 2020 [15]	F	71	anti-GBM + MPO	LMWH	14 days	GCs, CP, TPE, HD
Xu et al., 2020 [16]	M	59	anti-GBM	LMWH	12 days	GCs, CP, TPE, HD
Sugawara et al., 2017 [17]	M	32	anti-GBM	unspecified heparin	7 days	GCs, TPE, HD
Case 2 *	M	58	anti-GBM	UFH	10 days	GCs, CP, TPE
Case 4 *	F	74	anti-GBM + MPO	UFH	14 days	GCs, CP, TPE, HD

* Present cases. ANCA: anti-neutrophil cytoplasmic antibody, anti-GBM: anti-glomerular basement membrane disease, CP: cyclophosphamide, CVVH: continuous venovenous hemofiltration, ECMO: extracorporeal membrane oxygenation, GCs: glucocorticoids, HD: hemodialysis, LMWH: low-molecular-weight heparin, MPO: myeloperoxidase, PR3: proteinase 3, TPE: therapeutic plasma exchange, UFH: unfractionated heparin.

## Data Availability

The presented data are available in the patient’s medical records at the nephrology department where the patient was examined.
